# Assessment of three antiviral compounds against Borealpox virus infection in a mouse model

**DOI:** 10.1080/22221751.2026.2623694

**Published:** 2026-01-27

**Authors:** Jérémie Prévost, Nikesh Tailor, Angela Sloan, Kathleen Fulton, Sarah J. Medina, Jonathan Audet, Geoff Soule, David Safronetz

**Affiliations:** a Special Pathogens Program, National Microbiology Laboratory, Public Health Agency of Canada, Winnipeg, Canada; b Department of Medical Microbiology and Infectious Diseases, University of Manitoba, Winnipeg, Canada

**Keywords:** Borealpox, Orthopoxvirus, cidofovir, brincidofovir, tecovirimat, zoonotic disease

## Abstract

Borealpox virus (BRPV) is a zoonotic orthopoxvirus which was first documented in Alaska in 2015. Although most human infections are mild, a recent fatal case in an immunocompromised individual highlights the importance of studying this emerging pathogen. To date, less than 10 human cases of BRPV infection have been confirmed which limit the availability of clinical data on the effectiveness of antiviral therapy. Here, we examined the effectiveness of cidofovir, brincidofovir, and tecovirimat in cell culture and found all three were potent inhibitors of BRPV. In order to further study these modalities *in vivo*, we assessed immunocompetent and immunodeficient mice as disease models for BRPV. CAST/EiJ mice proved to be a suitable immunocompetent model for BRPV infection with high viral titres in several organs and route-dependent lethality. BRPV infection in immunodeficient mice, including STAT1^−/−^, *scid*, and NSG strains, was uniformly lethal and characterized by high viral titres as well as profuse fluid retention in the peritoneal cavity. Using CAST/EiJ mice, we then evaluated cidofovir, brincidofovir, and tecovirimat therapies. Post-exposure treatment resulted in significant reductions in viral titres and an improved clinical course of infection. Our results demonstrate the utility of mouse models to study the pathogenicity of BRPV and support the use of these antivirals to treat human infections.

## Importance

In humans, Borealpox virus (BRPV) is commonly associated with mild, self-limiting infections. However, in immunosuppressed individuals, infection can be severe and fatal. Due to a limited number of laboratory-confirmed cases and a lack of described animal models, little data on antiviral effectiveness against BRPV are available. Here we developed both immunocompetent and immunodeficient mouse models for the study of BRPV. In immunocompetent mice, BRPV infection resulted in high viral titres with organ-specific gross pathological abnormalities and route-dependant lethality. In immunodeficient mice, BRPV was uniformly lethal with varying times to lethal disease which appeared to coincide with viral replication. Notably, extensive fluid retention was also observed in these mice. Further assessment of cidofovir, brincidofovir, and tecovirimat therapy against BRPV infection demonstrated that all three compounds resulted in improved clinical condition and significant reductions in BRPV titres.

## Introduction

Borealpox virus (BRPV; formerly known as Alaskapox virus) is a zoonotic New World orthopoxvirus (OPXV) which was first clinically recognized in 2015 [1]. Since then, at least six additional cases have been diagnosed in individuals residing in or near Fairbanks, Alaska [2–6]. Prior to 2023–2024, documented human infections occurred in immunocompetent individuals and presented with ulcerated/umbilicated pustular plaques and surrounding erythema, fever, malaise, lymphadenopathy, and myalgia/arthralgia [2]. In these cases, symptoms resolved within a few weeks.

In 2024, BRPV garnered attention when it caused fatal disease in an immunocompromised 69-year-old man who lived in the remote Kenai Peninsula area of Alaska [5,6]. This individual had been undergoing anti-CD20 therapy for chronic lymphocytic leukaemia and was taking the oral antiviral medication acyclovir prophylactically when the first characteristic pox lesion appeared [5]. Due to delays in diagnosing BRPV infection, combination antiviral therapy including intravenous tecovirimat, intravenous vaccinia immunoglobulin, and oral brincidofovir, was not initiated until 2 months after symptom onset [5]. Despite his condition initially improving after one week of treatment, the patient later exhibited delayed wound healing, malnutrition, and acute renal and respiratory failure. At 138 days post-symptom onset, the patient succumbed to disease. Post-mortem examination revealed disseminated BRPV infection [2,5].

The ecology and geographic distribution of BRPV remains to be clarified, though small mammals, notably red-backed voles and shrews, have been implicated as potential zoonotic reservoirs with cats possibly serving as an intermediate host [2]. Although, the original six cases of BRPV infection all occurred in or near Fairbanks in individuals with no travel history, the recent fatal case was documented in a patient over 500 km away, implying a larger area of distribution. In North America, Northern red-backed voles (*Clethrionomys rutilus*) are common and have a geographic range that extends throughout Alaska and into the northern territories of Canada. Additionally, the closely related Southern red-backed vole (*Clethrionomys gapperi*) has a home range that extends throughout the rest of Canada and well into the central southern states of the USA [7]. Ecological studies are required to better elucidate viral prevalence in small mammals, but if in addition to Northern red-back voles, the Southern red-backed vole is involved in the enzootic lifecycle, the geographic distribution of BRPV could be substantial.

As an emerging OPXV, and in light of the recent global expansion of monkeypox virus (MPXV), it is paramount to assess the efficacy of potential medical countermeasures to treat BRPV infections. Due to the low frequency of clinical cases, this is best accomplished using a combination of *in vitro* and *in vivo* methodologies. To that end, in the current study, we evaluated both immunocompetent and immunodeficient mice as infection and disease models for BRPV. Our results suggest that like MPXV, CAST/EiJ mice are an appropriate pre-clinical evaluation model for BRPV. Using this model, we then explored the effectiveness of three anti-poxviral compounds to treat BRPV infection. Tecovirimat (TCV) is a small molecule inhibitor of OPXV replication that is FDA-approved drug for the treatment of human smallpox disease, and functions by targeting the viral phospholipase F13 (also known as VP37 or OPG057), which is required for the formation of mature virions [8–10]. Cidofovir (CDV) is a nucleotide analogue that inhibits viral DNA synthesis by targeting the viral DNA polymerase enzyme, while brincidofovir (BCV) is a lipid-conjugated metabolite of CDV that has greater bioavailability than its prodrug, implicating its potential to improve dosing and administration strategies [11,12]. Combined our results demonstrated that all three antivirals are effective in reducing BRPV titers, both *in vitro* and *in vivo*, and should be considered for use in treating BRPV infections in clinical settings.

## Methods

### Biosafety and animal ethics statement

All work with infectious BRPV was conducted in a biosafety level two laboratory using universal precautions and according to institutionally approved standard operating procedures by staff previously immunized with an Orthopoxvirus vaccine, as per the recommendations of an institutional risk assessment. Animal experiments were conducted at the National Microbiology Laboratory of the Public Health Agency of Canada. All experiments were approved by the institutional animal care committee per the guidelines of the Canadian Council of Animal Care. Invasive procedures were conducted on mice sedated with inhalational isoflurane. All efforts were made to minimize animal suffering and to reduce the number of mice utilized in these experiments. Animals were provided food and water *ad libitum* throughout the course of the experiments and were group housed in HEPA-filtered isolator units within a climate-controlled, dedicated room on a 12:12 light/dark cycle.

### Virus

Borealpox virus (Alaska_2015 strain, kindly provided by Dr. Sathesh Panayampalli, US Centres for Disease Control and Prevention) was propagated in Vero E6 cells and titered using a standard 50% tissue culture infectious dose (TCID_50_) assay methodology, essentially as previously described [13,14]. Prior to these experiments, viral stocks were characterized by deep sequencing and confirmed mycoplasma-free using an established molecular method [15]. The sequencing data used to confirm the stock virus sequence is publicly available on the SRA under BioProject PRJNA1370131 as BioSample SAMN53419813. Analysis revealed no differences in the stock virus used on these experiments compared with the previously deposited genome (accession number MN240300) [16].

### Antiviral compounds

Clinical grade CDV and TCV were obtained from Health Canada, whereas BCV was synthesized by MedChem Express. All three compounds were prepared as previously described [13,14].

### *In vitro* efficacy studies

Antiviral efficacy of TCV, CDV, and BCV were assessed using plaque reduction assays, essentially as previously described [13,14]. Briefly, nearly confluent monolayers of Vero E6 cells in 12-well plates were infected with 50 plaque-forming units (PFU) of BRPV for 1 h at 37°C and 5% CO_2_ with gentle rocking every 15 min. The inoculum was then removed and an overlay of MEM containing 2% FBS, 1% carboxymethyl cellulose and varying concentrations of CDV (0, 1, 3, 10, 30, 100 µM), BCV (0, 0.1, 0.3, 1, 3, 10 µM), or TCV (0, 0.1, 0.3, 1, 3, 10 µM) was added. Each condition was tested in duplicate in three independent experiments. After 7 days, 100 µl of 5 mg/mL MTT working solution was added to each well and incubated for 1 h at 37°C, 5% CO_2_. Plaques were counted and the titre in PFU/mL was used to calculate the half maximal inhibitory concentration (IC_50_) and the 90% maximal inhibitory concentration (IC_90_) for each compound by nonlinear regression analysis using GraphPad Prism software. Cell viability was assessed visually upon the addition of the MTT viability dye.

### *In vivo* modelling

Two independent assessments of CAST/EiJ mice were conducted. In the first iteration, 16 mice (male, 4–6 weeks old) were challenged with 1 × 10^5^ TCID_50_ of BRPV via intranasal (I.N.) instillation. In the second iteration, 16 age- and sex-matched mice were inoculated with 1 × 10^5^ TCID_50_ of BRPV via intraperitoneal (I.P.) injection. The challenge dose administrated represented the highest dose achievable based on tissue culture derived stocks and volume allowable for the I.N. route. For both experiments, mice were weighed daily and monitored for disease progression, with 3–4 mice per group euthanized at 5 and 10 days post-infection (DPI) for tissue collection and virological assessment. Tissue collections included lung, brain and nasal turbinates for the I.N. challenged mice and lung, liver, kidney, spleen, and brain for the I.P.-challenged mice. In addition, a superficial swab of the peritoneal cavity was performed to assess the presence of infectious virus. The remaining mice were monitored for survival until 28 DPI.

In a follow-up experiment, immunodeficient mice, including STAT1^−/−^ (B6.129S(Cg)-*Stat1^tm1Dlv^*/J), *scid* (CBySmn.Cg-*Prkdc^scid^*/J), and NSG (NOD.Cg-*Prkdc^scid^ Il2rg^tm1Wjl^*/SzJ) strains, were assessed as possible murine models of BRPV infection. For each strain, 16–21 mice (males, 4–6 weeks old) were inoculated with 1 × 10^5^ TCID_50_ of BRPV via I.P. injection and similarly monitored daily for disease progression. On days 5 and 8 (STAT1^−/−^ mice only), or days 5, 10, and 15 (*scid* and NSG mice only) post-infection, 4–5 mice per group were euthanized and tissues (lung, liver, kidney, and spleen) and a swab of the peritoneal cavity were collected for virological assessment.

Based on the results of the above experiments and to allow direct comparison to previous studies with MPXV, CAST/EiJ mice were selected for use in antiviral efficacy studies.

### *In vivo* efficacy studies

Four groups of 16 CAST/EiJ mice (males, 4–6 weeks old) were inoculated with 1 × 10^5^ TCID_50_ of BRPV via I.P. injection. Antiviral treatments were initiated at 1 DPI. TCV-treated mice received 100 mg/kg of compound delivered by oral gavage daily for 5 consecutive days. CDV-treated mice received 30 mg/kg of compound daily for 5 consecutive days by I.P. injection. BCV-treated mice received a loading dose of 10 mg/kg at 1 DPI and the maintenance doses of 5 mg/kg, every second day, all by oral gavage, until 7 DPI, for a total of four doses. The fourth group of mice were treated daily with placebo (diluent only) for 5 consecutive days, half via oral gavage and half via I.P. injection. Post-challenge, mice were monitored and weighed daily. At 10 DPI, 6 mice per group were exsanguinated and tissues collected for virological analysis. The remaining 10 mice per group were monitored for survival until 29 DPI.

### BRPV detection and titration

Viral DNA was extracted from 30 mg pieces of mouse tissues using the MagMAX viral/pathogen nucleic acid isolation kit on a KingFisher Apex instrument (both from Thermo Fisher Scientific). Molecular detection of BRPV was accomplished on a LightCycler 96 instrument (Roche) using TaqPath 1-step RT-qPCR reagents (Applied Biosystems) according to the manufacturer's specifications with BRPV-specific primers targeting the OPG001 gene (BRPV_FWD: CAAAATCTGTGGGCACTTGGTGA and BRPV_REV: CACCGGGGAGCAATCTGAAGA) and a double quench FAM probe (BRPV_PR: TTTCAGACCTCCACCGACGATGGCG) (all from IDT). Infectious BRPV titres were determined using standard TCID_50_ assays, as previously described [13,14]. Briefly, cryopreserved tissue specimens were thawed, mechanically homogenized, and clarified as described. For each specimen, a 10-fold serial dilution series was prepared and used to infect Vero E6 cells in triplicate wells of a 96 well plate. Plates were incubated for 7 days at 37°C, 5% CO_2_ after which the presence of cytopathic effect (CPE) was recorded and viral titers calculated using the Reed-Muench method [17].

### Serological detection of BRPV antibodies

Anti-BRPV antibodies were detected in serum collected from mice during timed necropsies or at the study endpoint using a standard ELISA technique. Briefly, individual wells of half area, high-binding 96-well plates (Corning) were coated with 50 ng of recombinant, purified truncated BRPV A-type inclusion protein (ATIp, Biomatik) diluted in 50 µl of phosphate-buffered saline (PBS) and stored at 4°C overnight. The following day, plates were washed three times with PBS containing 0.1% Tween 20 (PBS-T) on an automated plate washer (Biotek) and blocked for 1 h at room temperature (RT) with PBS-T supplemented with 5% skim milk. Plates were then washed again and fourfold serial dilutions of mouse serum prepared in PBS-T with 5% skim milk were added to duplicate wells and incubated at RT for 1 h. Following another wash, plates were incubated with an HRP-conjugated goat anti-mouse IgG (H + L) or goat anti-mouse IgM (mu) secondary antibodies (KPL) diluted 1:2000 in PBS-T with 5% skim milk, incubated for a further 1 hr at RT, and again washed as above. Finally, plates were incubated in the dark for 30 min at RT with TMB solution (Life Technologies) after which the optical density was measured at 650 nm (OD_650_) using a Synergy HTZ plate reader (Biotek). The area under the curve (AUC) was calculated from OD_650_ values obtained with serial dilutions for each sample. Cut-off values were established as the mean plus three times the standard deviation of the AUC for samples collected from naïve mice.

### Cytokine detection

Cytokine and chemokine responses were assessed in a serum from naïve, uninfected CAST/EiJ mice as well as samples collected at 10 DPI from mice enrolled in the *in vivo* efficacy study using a mouse 26-plex panel (EPX260-26088-901, Thermo Fisher Scientific) according to manufacturer instructions. Serum samples were diluted 1:4 and test plates were run using a Luminex MAGPIX instrument. The analytes examined included Eotaxin (CCL11), GM-CSF, GRO α (CXCL1), IFN-γ, IL-1β, IL-10, IL-12 (p70), IL-13, IL-17, IL-18, IL-2, IL-22, IL-23, IL-27, IL-4, IL-5, IL-6, IL-9, IP-10 (CXCL2), MCP-1 (CCL2), MCP-s (CCL7), MIP1α (CCL3), MIP-1β (CCL4), MIP-2α (CXCL2), RANTES (CCL5), TNF-α.

### Statistical analysis

Data were analysed using GraphPad Prism version 10.6.1 (GraphPad Software). Every data set was tested for statistical normality and this information was used to apply the appropriate (parametric or nonparametric) statistical test. Statistical details of experiments are indicated in the figure legends. *p* values < 0.05 were considered significant; significance values are indicated as ∗*p* < 0.05, ∗∗*p* < 0.01, ∗∗∗*p* < 0.001, ∗∗∗∗*p* < 0.0001.

## Results

### Characterization of BRPV infection in CAST/EiJ mice

**Figure 1.**
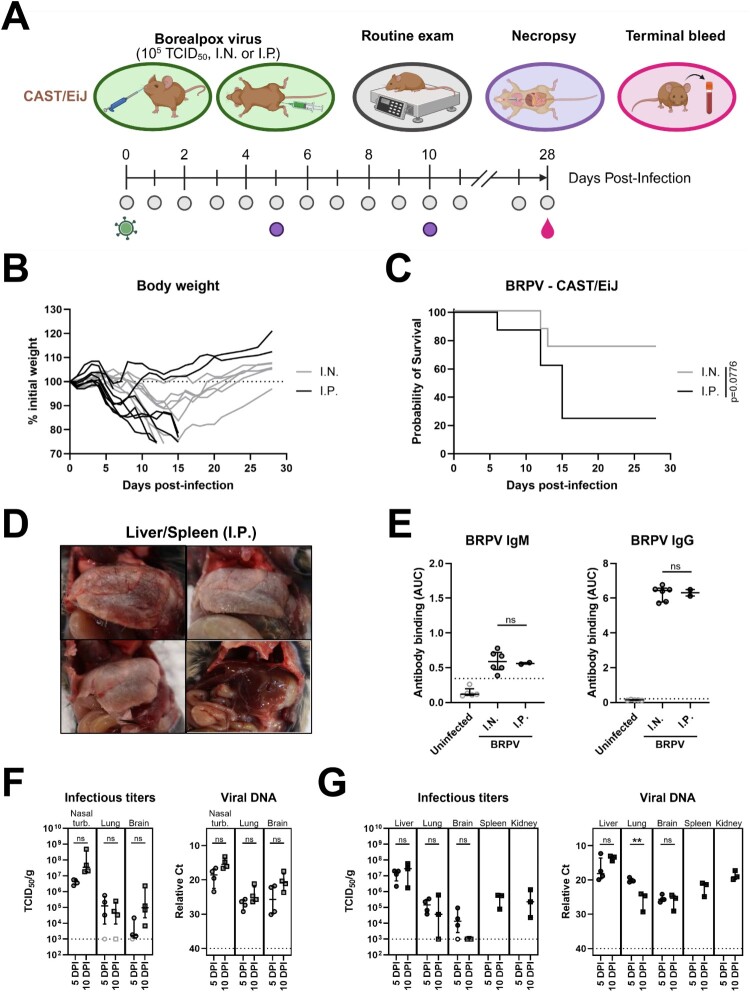
Assessment of CAST/EiJ mice as a model for Borealpox virus infection. CAST/EiJ mice were inoculated with 10^5^ TCID_50_ via intranasal instillation (I.N., *n* = 16) or intraperitoneal (I.P., *n* = 16) of Borealpox virus (BRPV, strain Alaska2015). At 5 and 10 DPI, three to four mice per group were euthanized and samples collected for virological and serological analysis. The remaining eight mice per group were monitored for disease progression. (**A**) Experimental layout. (**B**) Daily body weight assessment. (**C**) Survival plot. (**D**) Gross pathological abnormalities. Shown are representative images of spleens (showing splenomegaly) and livers (note the discoloration) collected from three terminally-ill animals inoculated via the I.P. route (top L and R, bottom L) and a healthy, non-infected control (bottom R). (**E**) Assessment of anti-BRPV IgM and IgG antibodies in serum collected from infected mice at 28 DPI. (**F**) Infectious viral titres and viral DNA detection in tissues collected at 5 and 10 DPI from I.N. inoculated mice. (**G**) Infectious viral titres and viral DNA detection in tissues collected at 5 and 10 DPI from I.P. inoculated mice. Dotted line in (**B**) represents 100% of the initial weight. (**E–G**) Dotted lines represent the limit of detection of the assays. Error bars represent medians +/- interquartile range (IQR). Open symbols represent undetectable values. Statistical significance was assessed using a Mantel–Cox logrank test in (**C**) or an unpaired *t* test or a Mann–Whitney *U* test in (**E–G**). **p < 0.01, ns: non-significant. AUC: area under the curve; BRPV: Borealpox virus; Ct: cycle threshold; DPI: days post-infection; I.N. intranasal; I.P. intraperitoneal; Nasal turb.: nasal turbinates; TCID_50_/g: 50% infectious dose per gram of tissue.

Based on the previous disease modelling studies for MPXV and other OPXVs [18–21], CAST/EiJ mice were first assessed as an infection model for BRPV (Figure 1A). Between 4–5 DPI, mice inoculated via I.N. or I.P. routes began showing signs of disease including weight loss (Figure 1B). Sharp declines in body weight were observed in mice in the I.P. group between 4–10 DPI. In contrast, mice in the I.N. inoculation group experience more gradual weight loss which coincided with increased survival rates (Figure 1B). Overall, six of eight (75%) mice in the I.P. challenge group and two of eight (25%) mice in the I.N. group succumbed to BRPV infection resulting in survival rates of 25% and 75%, respectively (Figure 1C). Signs of disease were consistent between the two inoculation groups, though onset and severity was delayed and slightly reduced in mice that received BRPV via I.N. instillation. In addition to weight loss, signs of disease included lethargy, unkempt fur, and respiratory manifestation which primarily displayed as rapid and shallow breathing. Upon necropsy of the mice in the I.P. group, gross pathological changes were observed in the liver (discoloration) and spleen (splenomegaly), which were absent in mice that were inoculated via I.N. instillation (Figure 1D). All surviving mice had detectable anti-BRPV IgM and IgG antibodies at the conclusion of the experiment (Figure 1E).

Viral titres were assessed by both molecular and infectious assays in organs collected at 5 and 10 DPI (Figures 1F–G). The greatest viral burdens in the I.N-challenged mice were observed in the nasal turbinates with titres increasing from 10^6^ TCID_50_/g at 5 DPI to greater than 10^7^ TCID_50_/g at 10 DPI (Figure 1F). Average lung titres dropped slightly between 5 and 10 DPI, whereas viral loads in the brain increased from essentially the infectious limit of detection (10^3^ TCID_50_/g) to an average of 10^5^ TCID_50_/g at 10 DPI. These results suggest that I.N. inoculation leads to local replication in the nose, but poor viral dissemination to neighbouring organs. On the other hand, mice challenged with BRPV via the I.P. route demonstrated a higher viral burden in liver samples with average titres of 10^7^ or greater TCID_50_/g, while lung titres were similar as those observed in the I.N. challenge group at both 5 and 10 DPI (Figure 1G). Although spleen and kidney samples were not available for assessment from the 5 DPI collection point, infectious virus was readily detectable in both organs at 10 DPI with titres of 10^5^ TCID_50_/g. Infectious titres in brain samples from mice inoculated via the I.P. route were lower than those from the I.N route, and infectious virus was not recovered from samples collected at 10 DPI, despite the detection of viral DNA. Since mice were not cardiac perfused prior to tissue collection, the discordant results between infectious assay and molecular tests, which was most evident in brain specimens, may be due to contaminating residual viral DNA from residual blood. Importantly, there was no evidence that neuroinvasion was involved in the disease progression of BRPV inoculated mice in this study. Free fluid in the peritoneal cavity was noticed in most terminally-ill mice and the presence of infectious virus within it was confirmed (see Figure S1A–B).

### Characterization of BRPV infection in immunodeficient mice

**Figure 2.**
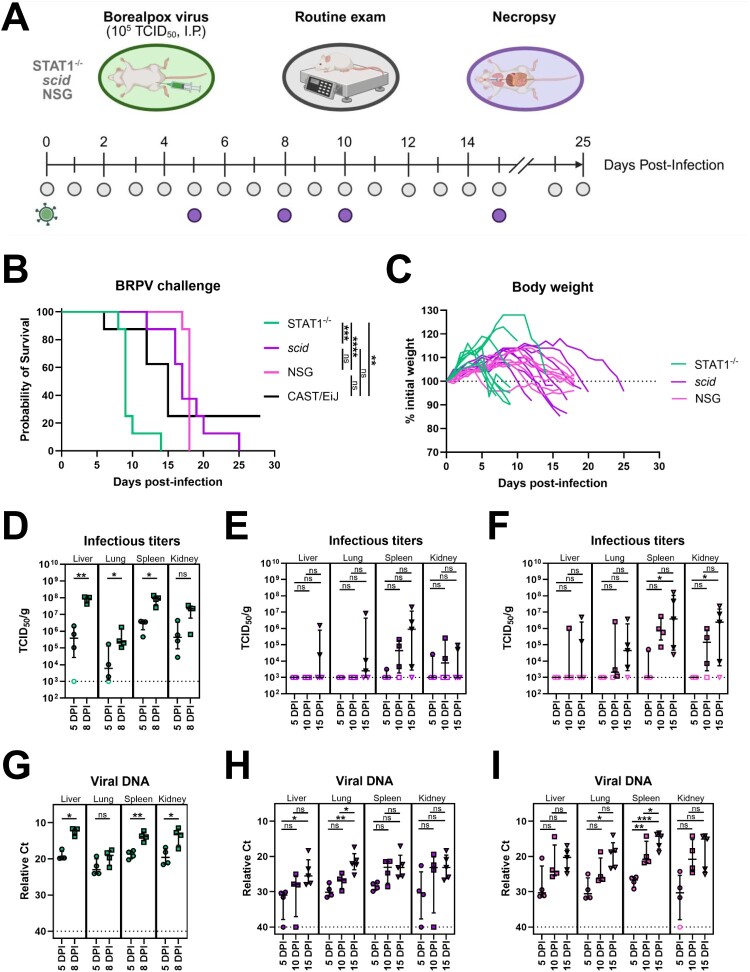
Assessment of immunodeficient mice as a model for Borealpox virus infection. Groups of 16–21 STAT1^−/−^, *scid*, and NSG mice were inoculated with 10^5^ TCID_50_ of Borealpox virus (BRPV, strain Alaska2015) via intraperitoneal injection. On days 5 and 8 (STAT1^−/−^ only), or days 5, 10, and 15 (*scid* and NSG only), 4–5 mice per group were euthanized and tissue collected for virological assessment, while the remaining eight mice per group were monitored for disease progression. (**A**) Experimental layout. (**B**) Survival plot. (**C**) Daily body weight assessment. (**D–F**) Infectious viral titers in tissues from (**D**) STAT1^−/−^, (**E**) *scid*, and (**F**) NSG mice. (**G–I)** Detection of viral DNA in tissues from (**G**) STAT1^−/−^, (**H**) *scid*, and (**I**) NSG mice. Dotted line in (**C**) represents 100% of the initial weight. (**D–I**) Dotted lines represent the limit of detection of the assays. Error bars represent medians +/– interquartile range (IQR). Open symbols represent undetectable values. Statistical significance was assessed using a Mantel–Cox logrank test in (**B**), an unpaired *t*-test or a Mann–Whitney *U* test in (**D,G**) and a Kruskal–Wallis test with a Dunn's post-test or a one-way ANOVA with a Holm–Sidak post-test in (**E–F, H–I**). *P < 0.05, **p < 0.01, ***p < 0.001, ****p < 0.0001, ns: non-significant. BRPV: Borealpox virus; Ct: cycle threshold; DPI: days post-infection; I.P.: intraperitoneal; TCID_50_/g: 50% infectious dose per gram of tissue.

To further assess BRPV infection within the context of an immunocompromised host, we evaluated disease progression in three immunodeficient strains of mice; STAT1^−/−^ (impaired IFN responses), *scid* (lacking T- and B cells), and NSG (lacking function T-, B-, and NK cells) (Figure 2A). Following I.P. challenge, BRPV infection was found to be lethal in all three strains of mice with median times to death of 9 DPI (range 8–14 DPI) in STAT1^−/−^ mice, 17 DPI (range 12–25 DPI) in *scid* mice, and 18 DPI (range 17–18 DPI) in NSG mice (Figure 2B). Among the three models, only the STAT1^−/−^ mice showed significant difference in time to death when compared to CAST/EiJ mice, succumbing to the infection 6 days earlier on average (Figure 2B). Similar signs of disease were noted in all three mice strains, including ruffled fur and lethargy which appeared 5–7 days prior to lethality. A hallmark of BRPV infection in immunodeficient mice was profuse fluid retention (sometimes more than 1 ml) in the peritoneal cavity (ascites) which masked weight loss, at least in the acute stages of infection (Figures 2C and S2A). Swab collections of the peritoneal fluid confirmed the presence of high titers of infectious BRPV (up to 10^6^ TCID_50_/ml) in all three immunodeficient mouse strains (see Figure S1A–B), correlating with viral titers observed in other major organs (Figure 2D–I). Gross pathology upon necropsy showed signs of hepatic damage (discoloration), including noticeable swelling of the gallbladder in most mice, while the other internal organs were mostly unremarkable (see Figure S2B). Interestingly, two pox-like lesions were noted on the tail of one NSG mouse (see Figure S2C). The difference in pathogenicity between the three immunodeficient models appears to be driven by distinct viral replication kinetics in tissues. Indeed, STAT1^−/−^ mice showed higher replication in all collected organs at earlier timepoints (10^5^–10^8^ TCID_50_/g at 8 DPI), while *scid* and NSG mice showed slow and steady increases in infectious titers and viral DNA, peaking peri-mortem at around 15 DPI (Figures 2D–I).

### Antiviral efficacy of cidofovir, brincidofovir, and tecovirimat *in vitro*

To initially assess if CDV, BCV, and TCV exhibit an antiviral effect on BRPV, we quantified the concentration of each compound that resulted in 50% and 90% inhibition of infectious virus, *in vitro*. As with other OPXVs, BRPV was sensitive to all three compounds (Table 1). Both TCV and BCV were highly effective at nanomolar concentrations, whereas CDV appeared less effective against BRPV, requiring mid-micromolar ranges for similar inhibitory effects. Compared to IC_50_ and IC_90_ values calculated for these antivirals using the same methodology against clade I and II MPXV isolates, BRPV appears to be less sensitive to TCV while the susceptibility to CDV and BCV were similar (Table 1) [13,14]. Although not directly measured quantitatively in the current study, cellular cytotoxicity was not observed during these experiments, which is expected since the concentrations of CDV, BCV, and TCV utilized herein were below previously recorded 50% cytotoxic concentrations (CC_50_) in African kidney cell lines [22–24].

**Table 1.** Inhibitory activity of Orthopoxvirus antivirals against Borealpox virus.

Virus	Strain	Tecovirimat		Cidofovir			Brincidofovir			Reference
		IC_50_ (µM)	IC_90_ (µM)	IC_50_ (µM)		IC_90_ (µM)	IC_50_ (µM)		IC_90_ (µM)	
BRPV	Alaska2015	0.381	0.707	25.57		104.3	0.578		1.059	This study
MPXV	Zaire1979 [clade I]	0.008	0.015	5.722		9.066	0.523		3.784	Warner et al. 2022 Prévost et al. 2024
	SP2833 [clade II]	0.006	0.012	3.827		12.83	0.149		0.231	Warner et al. 2022 Prévost et al. 2024
										

BRPV: Borealpox virus, MPXV: Monkeypox virus, IC_50_: 50% maximal inhibitory concentration, IC_90_: 90% maximal inhibitory concentration.

Warner et al. 2022 (PMID: 36318038).

Prévost et al. 2024 (PMID: 39243894).

### Antiviral efficacy of cidofovir, brincidofovir, and tecovirimat *in vivo*

**Figure 3.**
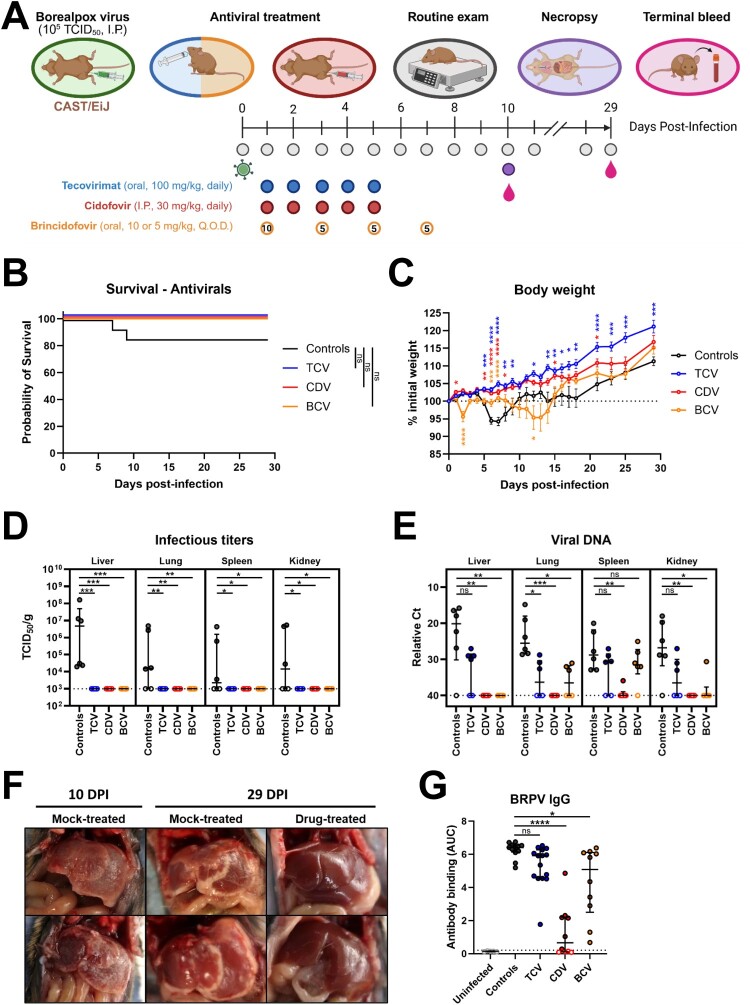
Treatment efficacy of tecovirimat, cidofovir, and brincidofovir against Borealpox virus infection in CAST/EiJ mice. Four groups of 16–21 CAST/EiJ mice were inoculated with 10^5^ TCID_50_ of Borealpox virus (strain Alaska2015) via intraperitoneal (I.P.) injection and treated with tecovirimat (TCV), cidofovir (CDV), brincidofovir (BCV), or placebo (diluent only). At 10 DPI, six mice per group were euthanized for virological assessments, while the rest were monitored for disease progression and survival. (**A**) Experimental layout. (**B**) Survival plot. (**C**) Daily body weight assessment. (**D**) Detection of infectious virus in tissues collected at 10 DPI. (**E**) Molecular detection of viral DNA in tissues collected at 10 DPI. (**F**) Comparison of gross pathological abnormalities in drug- versus mock-treated animals. Shown are representative images of livers (note the discoloration and scarring in the mock-treated group) collected at 10 and 29 DPI. The top row is images from mice which received placebo or CDV via I.P. injections, while the bottom row contains images from mice which received placebo or BCV via oral gavage. (**G)** Assessment of anti-BRPV IgG antibodies in serum collected from infected mice at 29 DPI. (**C**) Dotted line represents 100% of the initial weight. Error bars represent means +/– standard error of the mean (SEM). (**D,E,G**) Dotted lines represent the limit of detection of the assays. Error bars represent medians +/– interquartile range (IQR). Open symbols represent undetectable values. Statistical significance was assessed using a Mantel–Cox logrank test in (**B**), and a Kruskal–Wallis test with a Dunn's post-test or a one-way ANOVA with a Holm–Sidak post-test in (**C,D,E,G**). *p < 0.05, **p < 0.01, ***p < 0.001, ****p < 0.0001, ns: non-significant. BCV: brincidofovir; CDV: cidofovir; Ct: cycle threshold; I.P.: intraperitoneal; Q.O.D.: every other day; TCV: tecovirimat.

The antiviral efficacy of CDV, BCV, and TCV was further evaluated *in vivo* using the above described CAST/EiJ mouse model (I.P. inoculation, 10^5^ TCID_50_ BRPV challenge dose). Beginning at 1 DPI, mice were treated daily (CDV, TCV, placebo) or every second day (BCV) and monitored for disease progression (Figure 3A). As in the model development experiment, placebo-treated mice began showing signs of disease including weight loss between 4–5 DPI. However, disease severity appeared to be mitigated in part by the placebo treatments as suggested by weight loss not exceeding 10% (compared to 10–20% in the first experiment) and increased survival (80% compared to 25% in the first experiment) (Figures 3B–C). Nevertheless, treatments, most notably CDV and TCV, had a beneficial effect on disease progression as noted by essentially no weight loss in these groups (Figure 3C). BCV-treated mice experienced delayed weight loss, the extent of which was similar to that in placebo-treated mice, though they recovered quicker (Figure 3C). All drug-treated mice survived BRPV challenge. The most compelling evidence of an antiviral effect for these three compounds was observed in viral titers obtained from tissues collected at 10 DPI. Reduced levels of detectable viral DNA in tissues and peritoneal swabs from CDV-, TCV-, or BCV-treated mice was observed with no recovery of infectious virus from these animals (Figures 3D–E and S1C–D). By comparison, the majority of tissues collected from placebo-treated mice had detectable infectious virus, with liver specimens yielding the highest titers averaging between 10^6^–10^7^ TCID_50_/g (Figure 3D). Intriguingly, upon necropsy, notable liver gross pathology was observed in mock-treated animals both at 10 (timed necropsy) and 29 (survivors) DPI, which was absent in the drug-treated mice (Figure 3F).

**Figure 4.**
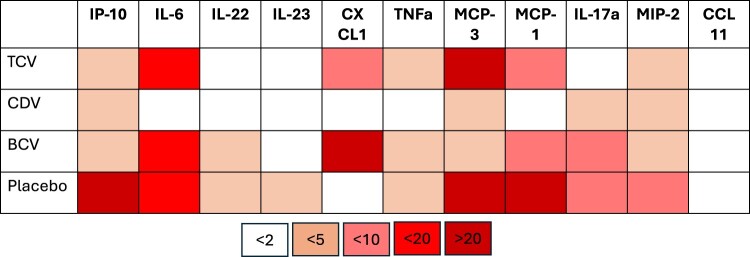
Comparison of host responses in serum from Borealpox infected mice treated with tecovirimat, cidofovir, brincidofovir, or placebo. CAST/EiJ mice were inoculated with 10^5^ TCID_50_ of Borealpox virus (strain Alaska2015) via intraperitoneal (I.P.) injection and treated with tecovirimat (TCV), cidofovir (CDV), brincidofovir (BCV), or placebo (diluent only). Sera collected at 10 DPI (*n* = 6 per group) was assessed for host responses using a Luminex-based multiplex kits. The heat map shows average fold-changes observed in sera from Borealpox virus infected and treated mice compared to uninfected controls.

Eleven of the 26 analytes assessed in the Luminex-based cytokine/chemokine assay cross-reacted with CAST/EiJ mouse sera and demonstrated that host innate responses were mostly dampened in treated mice, particularly in those receiving CDV whose responses most closely mirrored those from uninfected animals (Figure 4, Supplemental file). Placebo-treated, infection control, mice had notable (>20 fold) increases in inflammatory and immune cell recruitment chemokines including IP10, MCP-1 and MCP-3 which, with the exception of MCP-3 levels in the TCV treatment group, did not exceed eightfold increases in treated mice when compared to the uninfected controls. Other notable observations included increased detection in IL-6 in all infected mice with the exception of those receiving CDV and increased levels of CXCL1 in TCV- and BCV-treated mice. Anti-BRPV IgG antibodies were detected in most mice that survived challenge, though significantly less specifically-reactive antibodies were detected in mice which were treated with CDV compared with control (placebo-treated) mice, as well as those receiving TCV or BCV (Figure 3G).

## Discussion

The recent global expansion of MPXV has led to increased awareness of OPXVs and their impact on public health. Declining immunity in human populations following the cessation of the smallpox immunization program, climate change and increased human–wildlife interactions are providing increased opportunity for spillover events of OPXVs and other zoonotic pathogens [25]. The first case of BRPV (then referred to as Alaskapox) was identified in 2015 [1]. Since then, less than 10 cases have been confirmed, most of which were mild [2]. However, a recent fatal case in an immunocompromised individual highlights the importance of studying BRPV and assessing antivirals to limit infection in uniquely susceptible populations who are at risk of an increasingly severe disease [5].

Limited data exist on treatment efficacy for clinical cases of BRPV infection. Due to the self-limiting nature of these infections as well as the limited diagnostic support available at the time, treatment consisted primarily of supportive care and antibiotics to mitigate secondary bacterial infections [2]. Laboratory confirmation of BRPV infection in the single fatal case was substantially delayed; however, once confirmed, the administration of intravenous TCV and vaccinia immunoglobulins was initiated as well as oral BCV. Although an initial improvement was observed, the patient ultimately succumbed to the infection [5]. The purpose of the pre-clinical experiments presented here was to address the gap in knowledge with respect to the antiviral efficacy of TCV and BCV, as well as CDV, both *in vitro* and *in vivo*.

In a first step, mouse models for *in vivo* efficacy studies needed to be evaluated. CAST/EiJ mice have previously been used for human pathogenic OPXVs, including several assessments against historic and emerging lineages of MPXV [13,14,18–21]. Post-BRPV infection, these mice developed similar viral loads and signs of disease as those infected with MPXV, particularly MPXV clades I and IIa, which included weight loss of approximately 20% (Figure 1B). Upon necropsy, gross pathological abnormalities were also evident and included splenomegaly and discoloration of the liver (Figure 1D). Lethality was route-dependant with mice challenged via the I.N. route mostly survived BRPV infection, while those infected via the I.P. route mostly succumbed (Figure 1C). Although the I.P. challenge route does not mimic natural exposure and circumvents the potential influence of mucosal immunity and other factors that may play a role in natural infection, it offers a convenient challenge model. Combined, these initial studies suggested that, as with MPXV, CAST/EiJ mice were a suitable model with which antiviral efficacy could be evaluated.

In addition to evaluating immunocompetent mice, and based on the fatal case in an immunocompromised patient, we sought to evaluate three immunodeficient mouse strains: NSG mice which lack functional T-, B-, and NK cells; *scid* mice which lack T- and B cells; and STAT1^−/−^ mice which have impaired IFN responses. BRPV infection was lethal in all three strains with varying times to lethal disease (Figure 2B). Lethality appeared to correlate with viral burden (Figures 2D–I). In STAT1^−/−^ mice, BRPV achieved systemic infection with high organ titers by 8 DPI and was uniform lethality within 14 DPI (Figures 2D and G). In contrast, viral loads in NSG and *scid* mice were delayed beyond 10 DPI with lethality similarly delayed (Figures 2E–F, H–I). Although weight loss in these mouse strains was indicator of ensuing lethality, it was masked in the acute stages of infection by fluid retention and build up in the peritoneal cavity (see Figure S2A). Combined, the observations made during these experiments in immunodeficient mouse strains seem to mirror those of the immunosuppressed patient who ultimately succumbed to BRPV infection. The rapid and unchecked viral replication leading to death in STAT1^−/−^ mice suggest that an initial innate immune response is essential to controlling BRPV infection. However, without functional T- and/or B cells, BRPV infection will persist and eventually overwhelm the host, as was observed in the *scid* and NSG models. Corroborating these findings, earlier work with CAST/EiJ mice, which are wild-derived but inbred derivatives of *Mus musculus castaneus*, suggest their unique susceptibility to infection with MPXV and vaccinia virus (and presumably other OPXVs) is due to an inadequate innate and cellular response when compared to BALB/c mice [26]. Further examination of the mechanism leading to the abundant fluid build-up is required in immunodeficient mouse strains and their corresponding wildtype counterparts, but our data suggests that hepatic or renal dysfunction may be occurring in these mice strains, the latter of which was documented in the fatal human case [2,6]. However, our observations could also be attributed to inflammatory exudation from severe viral peritonitis caused by the I.P. challenge route.

In Vero cell culture TCV, BCV, and CDV all exhibited inhibitory effects on BRPV (Table 1). The IC_50_ and IC_90_ values for BCV observed in the current study are similar to those reported for other OPXVs, though for TCV these same values were approximately 60-fold higher than those observed for MPXV. This phenotype could be related to natural polymorphisms in the TCV binding site of the BRPV F13 protein [10]. Alignment of the F13L gene of BRPV revealed three mutations at locations known to produce TCV resistance in MPXV, but the mutations in BRPV are not to a known resistance genotype (see Table S1). The IC_50_ and IC_90_ values for CDV against BRPV were 5–10 fold higher than the same values for MPXV; however, the values observed here were similar to those measured against other OPXVs including vaccinia, cowpox, ectromelia, and rabbitpox viruses, albeit in different cell lines [27–30]. Nevertheless, the encouraging antiviral effects observed in cell culture supported further evaluation of these modalities in a mouse model. Based on the uniform disease progression, the systemic and high titre viral loads and the lethality noted in the pilot studies, CAST/EiJ mice were used for *in vivo* assessments of the three compounds.

Using routes indicative of human therapeutic administration, TCV and BCV were given orally, daily or every second day, respectively, via a feeding tube. Although in the fatal human case of BRPV infection, TCV was administered intravenously, the oral route is preferred unless the infection is advanced and the patient is unable to tolerate oral delivery (e.g. if renal impairment is suspected). CDV was given daily via I.P. injections, a surrogate for intravenous administration. Interestingly, mock-treated mice, which received diluent only either by oral gavage or I.P. injection, showed noticeable symptom improvement compared to untreated mice, with only 25% succumbing to infection and disease progression characterized by minimal (approximately 6%) weight loss (Figures 3B–C). The apparent beneficial effect of supportive care in mouse models of viral infection has been well described elsewhere, including in Ebola virus models where increases of up to 30% in infected mice have been observed following PBS treatments [31]. We hypothesize this may be facilitated in part by fluid replacement. Despite the increased survival rates in this group, post-exposure delivery of TCV, CDV, or BCV improved the clinical outcome of BRPV infection, with mice in the TCV and CDV groups showing no signs of disease. Mice treated with BCV experienced slight (<5%) weight loss which was delayed in comparison to the controls (Figure 3C). Host immune responses, as measured by fold-changes in selected cytokine and chemokine levels in infected and treated animals, compared with uninfected controls, were generally less pronounced in mice receiving specific antiviral therapy, most notable in CDV-treated mice (Figure 4). Infectious virus was not recovered from any of the drug-treated mice, whereas all control (placebo) treated mice had infectious virus detected in at least liver specimens, with most also having detectable viral loads in lung, spleen, and kidney samples (Figure 3D). Viral DNA was documented in all but one tissue specimen from control mice as well as from most TCV- and BCV-treated mice (Figure 3E). In contrast, tissues collected from CDV-treated mice were uniformly negative for viral DNA, with the exception of a single spleen specimen (Figure 3E). Also notable, and in support of the antiviral effect these modalities had on clinical outcome, gross pathological abnormalities were observed at both 10 and 29 DPI in liver samples from placebo-treated animals, but not in any of the BCV, CDV, or TCV treatment groups (Figure 3F).

With only a few clinical cases recognized to date, much remains to be learned about BRPV, its incidence in humans, and the overall region of endemicity. Its potential rodent reservoirs, such as Northern red-backed voles, masked shrews, northern flying squirrels, and North American red squirrels are widely distributed across northern areas of North America [2], which means that BRPV could be found in the wild in a broader area than previously thought. While current evidence of human-to-human transmission is lacking for BRPV, the possibility of genetic mutations leading to increased transmissibility (and perchance increased virulence) cannot be ignored. Although as a double-stranded DNA virus, this may seem unlikely, this prospect was realized during the recent global mpox outbreak [32]. The data presented here can serve as a benchmark for further clinical evaluation of these three antivirals in confirmed cases of BRPV infection. Further, the mouse models described herein provide a standard with which future studies may be conducted. As with other OPXVs, the CAST/EiJ model appears suitable for further evaluation of medical countermeasures including therapeutics and vaccines, whereas the immunodeficient models provide a mechanism with which further pathogenesis studies can be conducted to provide insight into BRPV infections in high risk human populations.

## Supplementary Material

Table S1.docx

BRPV cytokine raw data.xlsx

SUPPL FIGS BRPV.pdf
