# Supplementary material for: Assessment of three antiviral compounds against Borealpox virus infection in a mouse model
Source: Emerg Microbes Infect. 2026 Jan 27;15(1):2623694. doi: 10.1080/22221751.2026.2623694 (PMC12915423; doi:10.1080/22221751.2026.2623694)
Supplement: Table S1.docx [file TEMI_A_2623694_SM1186.docx]

| Known resistance marker in MPXV | BRPV sequence | MPXV Reference |
| --- | --- | --- |
| A295E | A295V | Garrigues et al. 2023 |
| I372N | I372K | Garrigues et al. 2023 |
| - | C-terminal end has a 3 a.a. insertion | - |

Supplemental Table S1. Comparison of known TCV resistance mutations in the F13L gene of MPXV and BRPV

Garrigues et al. 2023. PMID: 37338408
