# Supplementary material for: Assessment of three antiviral compounds against Borealpox virus infection in a mouse model
Source: Emerg Microbes Infect. 2026 Jan 27;15(1):2623694. doi: 10.1080/22221751.2026.2623694 (PMC12915423; doi:10.1080/22221751.2026.2623694)
Supplement: SUPPL FIGS BRPV.pdf [file TEMI_A_2623694_SM1184.pdf]

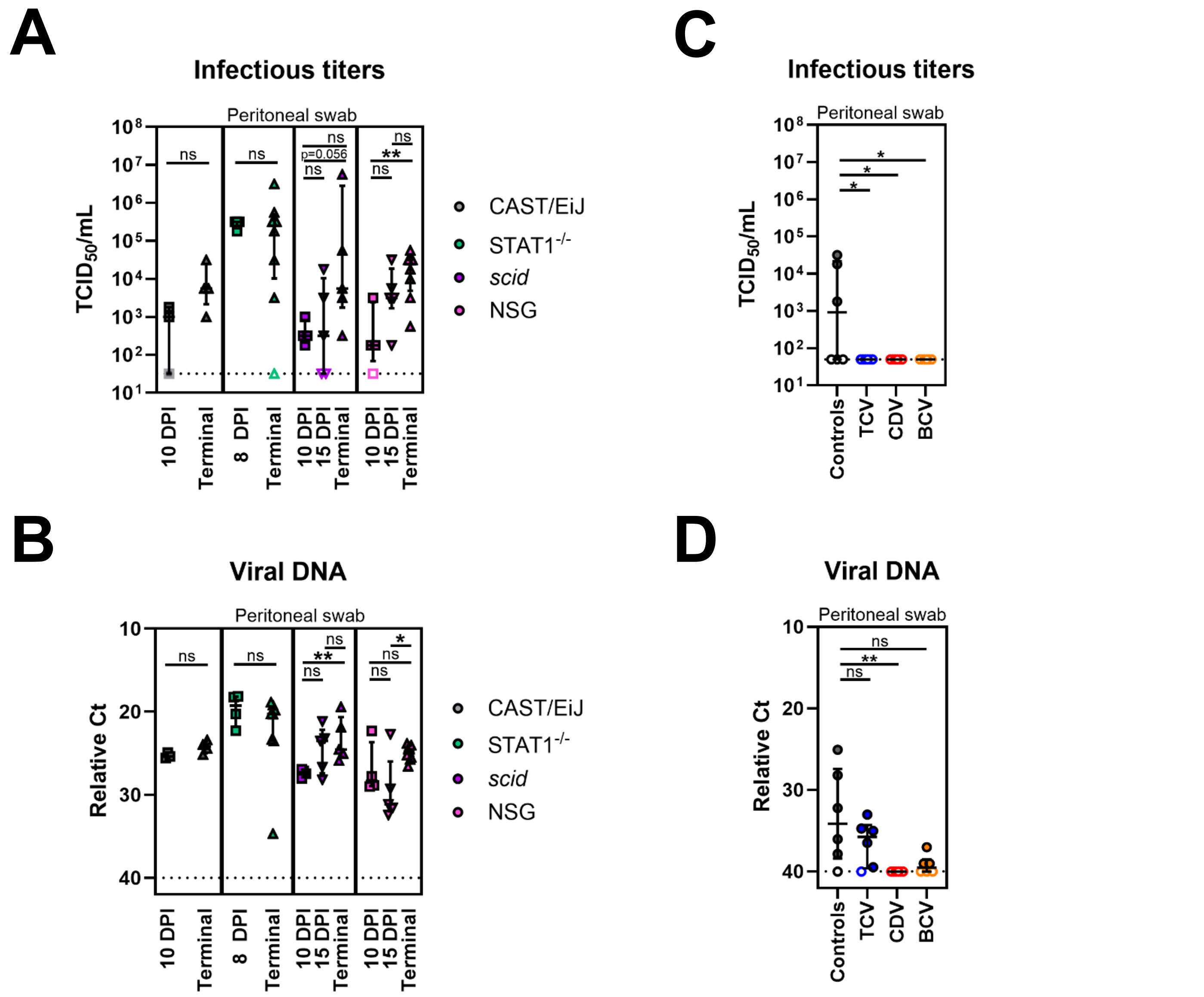

Figure S1. Detection of Borealpox virus in the peritoneal cavity of infected mice.
(**A-B**) Groups of CAST/EiJ, STAT1$^{-/-}$, *scid*, and NSG mice were inoculated with 10^5 $TCID_{50}$ of Borealpox virus (strain Alaska2015) via intraperitoneal injection. On days 5, 8, 10, 15 or on humane endpoint (terminal), mice were euthanized, and swabs of the peritoneal cavity were collected for quantification of (**A**) infectious viral titers and (**B**) viral DNA. (**C-D**) CAST/EiJ mice were inoculated with 10^5 $TCID_{50}$ of Borealpox virus (strain Alaska2015) via intraperitoneal injection and further treated with placebo (controls), tecovirimat (TCV), cidofovir (CDV) or brincidofovir (BCV). On day 10, mice were euthanized, and swabs of the peritoneal cavity were collected for quantification of (**C**) infectious viral titers and (**D**) viral DNA. Dotted lines represent the limit of detection of the assays. Error bars represent medians +/- interquartile range. Open symbols represent undetectable values. Statistical significance was assessed using (**A-B**) an unpaired t test or a Mann-Whitney U test and (**C-D**) a Kruskal-Wallis test with a Dunn's post-test. *P < 0.05, **P < 0.01, ns: non-significant. Ct: cycle threshold

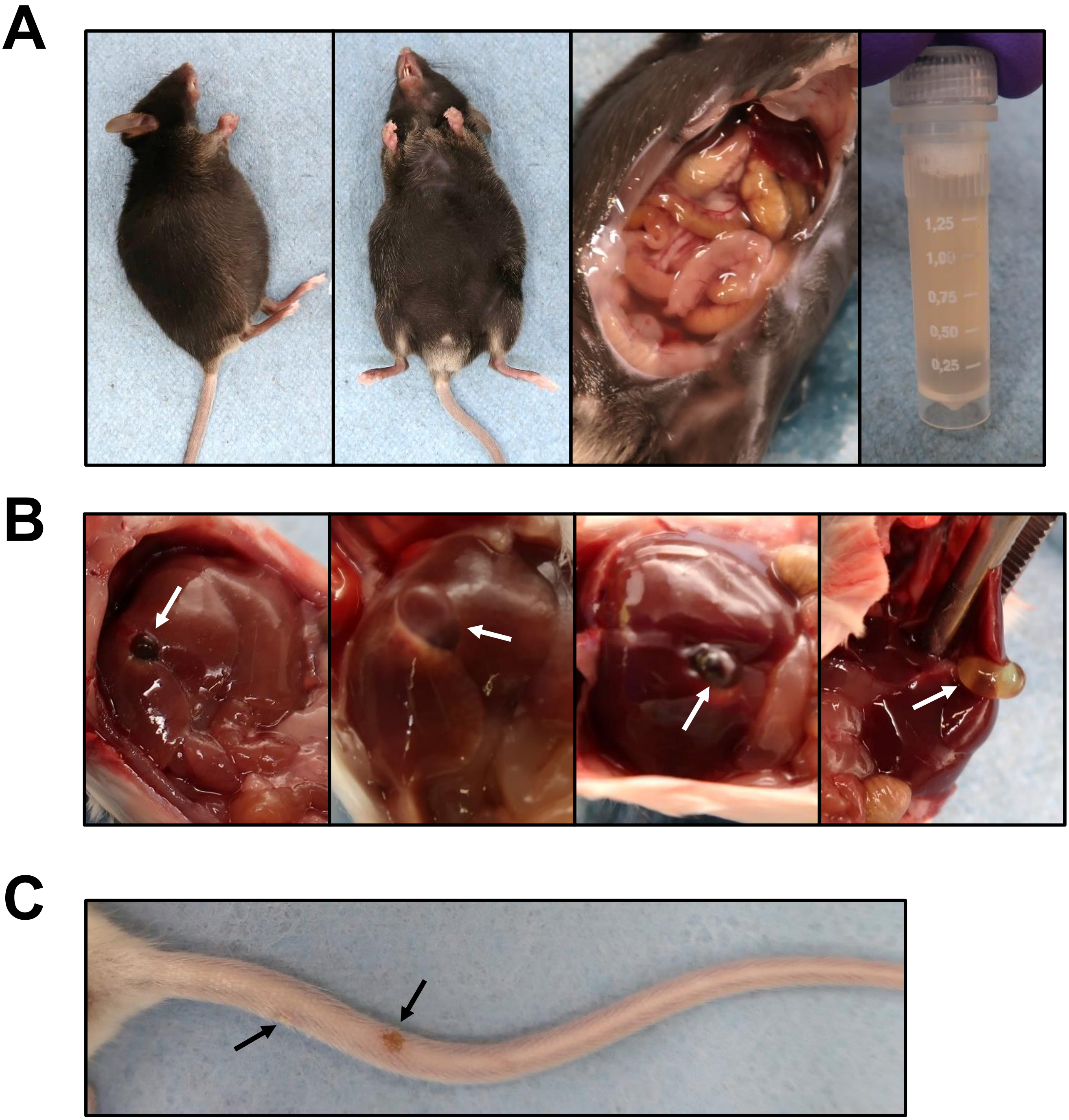

Figure S2. Gross pathology of immunodeficient mice infected with Borealpox virus.
STAT1$^{-/-}$, *scid*, and NSG mice were inoculated with 10^5 $TCID_{50}$ of Borealpox virus (strain Alaska2015) via intraperitoneal injection. Upon reaching human endpoint, mice were euthanized and subject to necropsy to identify gross pathology. (**A**) Images from STAT1$^{-/-}$ mice showing fluid retention in their abdominal cavity. (**B**) Images from *scid* mice showing swelling of their gallbladder (white arrows). (**C**) Images from a NSG mouse showing pox-like lesions on its tail (black arrows).
